# User requirements for quantitative radiological reports in multiple sclerosis

**DOI:** 10.1007/s00330-025-11544-x

**Published:** 2025-04-16

**Authors:** David R. van Nederpelt, Zoe C. Mendelsohn, Lonneke Bos, Rozemarijn M. Mattiesing, Olga Ciccarelli, Jaume Sastre-Garriga, Ferran Prados Carrasco, Joost P. A. Kuijer, Hugo Vrenken, Joep Killestein, Menno M. Schoonheim, Bastiaan Moraal, Tarek Yousry, Giuseppe Pontillo, Àlex Rovira, Eva M. M. Strijbis, Bas Jasperse, Frederik Barkhof, on behalf of the Magnetic Resonance Imaging in Multiple Sclerosis study group, Olga Ciccarelli, Jaume Sastre-Garriga, Menno M. Schoonheim, Àlex Rovira, Frederik Barkhof, Ahmed T. Toosy, Massimo Filippi, Christian Enzinger, Claudio Gasperini, Cristina Granziera, Nicola De Stefano, Gabriele De Luca, Maria A. Rocca

**Affiliations:** 1 MS Center Amsterdam, Radiology and Nuclear Medicine, Amsterdam Neuroscience, Amsterdam UMC location VUmc, Amsterdam, The Netherlands; 2 Centre for Medical Image Computing, Department of Medical Physics and Biomedical Engineering, University College London, London, UK; 3 Department of Radiology, Charité School of Medicine and University Hospital Berlin, Berlin, Germany; 4 Department of Neuroinflammation, Queen Square MS Centre, UCL Queen Square Institute of Neurology, Faculty of Brain Science, University College of London, London, UK; 5 NIHR University College London Hospitals Biomedical Research Centre, London, UK; 6 Department of Neurology, Multiple Sclerosis Centre of Catalonia, Hospital Universitari Vall d’Hebron, Barcelona, Spain; 7 e-Health Center, Universitat Oberta de Catalunya, Barcelona, Spain; 8 MS Center Amsterdam, Neurology, Amsterdam Neuroscience, Amsterdam UMC location VUmc, Amsterdam, The Netherlands; 9 MS Center Amsterdam, Anatomy and Neuroscience, Amsterdam Neuroscience, Amsterdam UMC location VUmc, Amsterdam, The Netherlands; 10 Lysholm Department of Neuroradiology and the Neuroradiological Academic Unit, Department of Brain Repair and Rehabilitation, University College London Hospitals NHS Foundation Trust National Hospital for Neurology and Neurosurgery, London, UK; 11 Departments of Advanced Biomedical Sciences and Electrical Engineering and Information Technology, University of Naples Federico II, Naples, Italy; 12 Section of Neuroradiology, Department of Radiology, Hospital Universitari Vall d’Hebron, Universitat Autònoma de Barcelona, Barcelona, Spain; 13 Queen Square Multiple Sclerosis Centre, Department of Neuroinflammation, UCL Institute of Neurology, London, United Kingdom; 14 Neuroimaging Research Unit, Division of Neuroscience, Istituto di Ricovero e Cura a Carattere Scientifico, San Raffaele Scientific Institute, Milan, Italy; 15 Neurorehabilitation Unit, Istituto di Ricovero e Cura a Carattere Scientifico, San Raffaele Scientific Institute, Milan, Italy; 16 Neurology Unit, Istituto di Ricovero e Cura a Carattere Scientifico, San Raffaele Scientific Institute, Milan, Italy; 17 Department of Neurology, Medical University of Graz, Graz, Austria; 18 Division of Neuroradiology, Vascular and Interventional Radiology, Department of Radiology, Medical University of Graz, Graz, Austria; 19 Department of Neurosciences, S. Camillo-Forlanini Hospital, Roma, Italy; 20 Translational Imaging in Neurology (ThINK) Basel, Department of Biomedical Engineering, Faculty of Medicine, University Hospital Basel and University of Basel, Basel, Switzerland; 21 Department of Neurology, University Hospital Basel, Basel, Switzerland; 22 Research Center for Clinical Neuroimmunology and Neuroscience Basel (RC2NB), University Hospital Basel and University of Basel, Basel, Switzerland; 23 Department of Medicine, Surgery and Neuroscience, University of Siena, Siena, Italy; 24 Nuffield Department of Clinical Neurosciences, Oxford University, Oxford, UK

**Keywords:** Multiple sclerosis, Magnetic resonance imaging, Consensus, Radiology

## Abstract

**Objectives:**

Quantitative radiological reports (QReports) can enhance clinical management of multiple sclerosis (MS) by including quantitative data from MRI scans. However, the lack of consensus on the specific information to include, on and clinicians’ preferences, hinders the adoption of these imaging analysis tools. This study aims to facilitate the clinical implementation of QReports by determining clinicians’ requirements regarding their use in MS management.

**Materials and methods:**

A four-phase Delphi panel approach was employed, involving neurologists and (neuro)radiologists across Europe. Initial interviews with experts helped develop a questionnaire addressing various QReport aspects. This questionnaire underwent refinement based on feedback and was distributed through the MAGNIMS network. A second questionnaire, incorporating additional questions, was circulated following a plenary discussion at the MAGNIMS workshop in Milan in November 2023. Responses from both questionnaire iterations were collected and analyzed, with adjustments made based on participant feedback.

**Results:**

The study achieved a 49.6% response rate, involving 78 respondents. Key preferences and barriers to QReport adoption were identified, highlighting the importance of integration into clinical workflows, cost-effectiveness, educational support for interpretation, and validation standards. Strong consensus emerged on including detailed lesion information and specific brain and spinal cord volume measurements. Concerns regarding report generation time, data protection, and reliability were also raised.

**Conclusion:**

While QReports show potential for improving MS management, incorporation of the key metrics and addressing the identified barriers related to cost, validation, integration, and clinician education is crucial for practical implementation. These recommendations for developers to refine QReports could enhance their utility and adoption in clinical practice.

**Key Points:**

***Question*** *A lack of consensus on essential features for quantitative magnetic resonance imaging reports limits their integration into multiple sclerosis management.*

***Findings*** *This study identified key preferences, including detailed lesion information, specific brain and spinal cord measurements, and rigorous validation for effective quantitative reports.*

***Clinical relevance*** *This study identified essential features and barriers for implementing quantitative radiological reports in multiple sclerosis management, aiming to enhance clinical workflows, improve disease monitoring, and ultimately provide better, data-driven care for patients through tailored imaging solutions.*

**Graphical Abstract:**

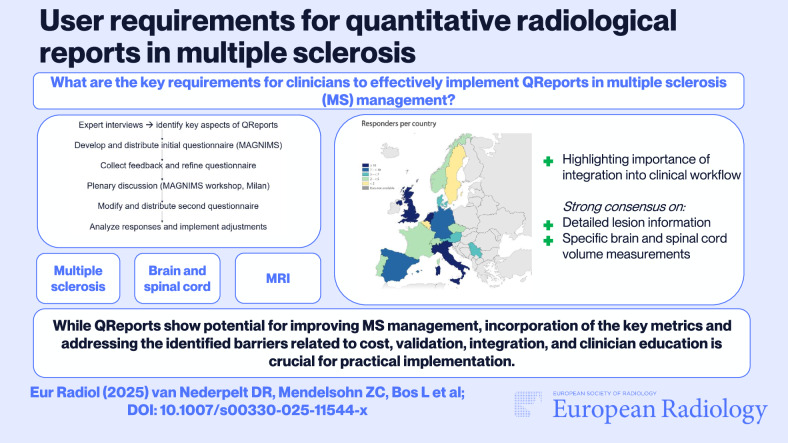

## Introduction

Multiple sclerosis (MS) is a chronic neurological condition characterized by a wide spectrum of symptoms often leading to progressive and irreversible disability [1]. Magnetic resonance imaging (MRI) plays a crucial role in the diagnosis and monitoring of patients with MS by visualizing lesions and structural changes in the brain and spinal cord [2]. Over the past three decades, advances in automated lesion and brain volume segmentation techniques have revolutionized the analysis of MR images. These automated methods offer increased sensitivity, accuracy, reproducibility, and significantly reduced processing time compared to manual segmentation [3–6], but have almost exclusively been used for research purposes.

In recent years, these automated segmentation methods have been adapted for the clinical management of MS by the development of both commercial and non-commercial quantitative radiological reports (QReports) [7]. QReports offer automated quantification of brain and lesion volumes contextualized by accompanying (normative) reference data. These provide clinicians and patients with potentially valuable information that can be used to support the diagnosis, prognosis, and disease monitoring [8]. However, despite the availability of several QReports, few have obtained widespread clinical implementation, possibly due to a lack of alignment with the specific requirements and preferences of clinicians for smooth and meaningful implementation in daily clinical routine, which remain understudied.

Using a Delphi panel method, this study addressed this literature gap by synthesizing a consensus among expert MS clinicians (neurologists and radiologists) regarding user requirements for QReports in MS. Through systematic identification of user requirements, we will provide recommendations that can guide the development of QReports that will improve the quality of care for patients with MS.

## Methods

The Delphi panel method was used to establish a consensus of QReport recommendations [9]. Our approach consisted of four sequential phases and three distinct rounds of a questionnaire.

### Preparatory phase

In the first phase, topics for the questionnaire were established through semi-structured interviews with three neurologists and three neuroradiologists from the MS Center Amsterdam. The questionnaire was developed online using Google forms (Google.com) and systematically addressed the use for diagnosis, monitoring, and prognosis purposes for the relevant MRI measures identified in the semi-structured interview.

### Phase 1

Members for an advisory board were recruited from the MAGNIMS Study Group (MAGNIMS (Magnetic Resonance Imaging in MS)) to review the questionnaire and provide their expert opinions on topics and questions to in- or exclude. This group consisted of four neuroradiologists, two neurologists, and four researchers who were actively involved in the MS-MRI field, and located in the Netherlands, the United Kingdom, and Spain. The following topics were included in the questionnaire: (1) QReport measurements, (2) QReport visualization, (3) clinical workflow, (4) deployment procedure, (5) scanners and sequences, (6) validation and testing, (7) quality control, (8) patient involvement. This resulted in a total of 63 questions that were sent out. Clinicians were asked questions for three different scenarios: diagnosis, prognosis, and monitoring. For each question, the answers were either Yes/No or on a 5-point scale: strongly agree, agree, neutral, disagree, and strongly disagree. Every question also had an “I don’t know” option. The “I don’t know” option was included to mitigate the influence of (self-assessed) lack of appropriate knowledge. Four open questions were included. The final questionnaire is provided in the supplementary materials.

### Phase 2

Before the review of the second phase, consensus was defined as at least 50% agreement. Using the Likert scale, agreement was defined as “agree” or “strongly” agree answers. Good agreement was defined as over 75% agreement, and moderate agreement as above 50% agreement, based on previous peer-reviewed studies [9]. User requirements, according to questionnaire responses and comments, were classified as highly desirable (≥ 75% agreement), desirable (≥ 50% < 75% agreement) and currently not desired (< 50% agreement). Responses containing “I don’t know” were excluded from the agreement calculations. A table of the percentage response rate per question is provided in Supplementary Table 1.

#### Round 1

For the first round of the second phase, the invitation for the questionnaire was disseminated among recipients of the MAGNIMS email list (*n* = 155). Recipients were encouraged to share the questionnaire with other clinicians active in the MS field, also outside of MAGNIMS. Open text boxes were provided after each section for responders to provide suggestions or comments on the questions or general comments. Additionally, a final overall suggestion box was provided at the end of the questionnaire. Responders were instructed to base their answers on their clinical needs rather than from a research perspective.

#### Round 2

After the first round, feedback from responders was collected. The topics and questions included in the second iteration of the questionnaire were determined based on the responses from the first round of the questionnaire. Additionally, input from an anonymous survey conducted during the MAGNIMS Study Group meeting in November 2023 was considered, which was based on the responses and comments on the first questionnaire.

#### Round 3

Subsequently, a revised and abbreviated version of the questionnaire, consisting of 19 questions, was circulated among the same group of (neuro)radiologists and neurologists after approval by the advisory board. In this follow-up iteration, questions in round 1 that achieved consensus were removed, items requiring clarification were revised, and additional topics were included based on survey feedback. These additional topics consisted of, among others, the inclusion of spinal cord measurements and the current QReport landscape among responders. Responders were reminded through email with a maximum of 3 reminders over a span of 1.5 months. A total of 62 (79.5% of the original responders) responded to the second questionnaire. For this questionnaire, a second table of the percentage response rate per question is provided in Supplementary Table 2.

### Phase 3

Responses to the two iterations of the questionnaire were collected and processed. The advisory board made adjustments to items when needed, considering the feedback from participants in the Delphi panel rounds and the comments received throughout the process.

## Results

### Responder demographics and response rate

**Fig. 1:**
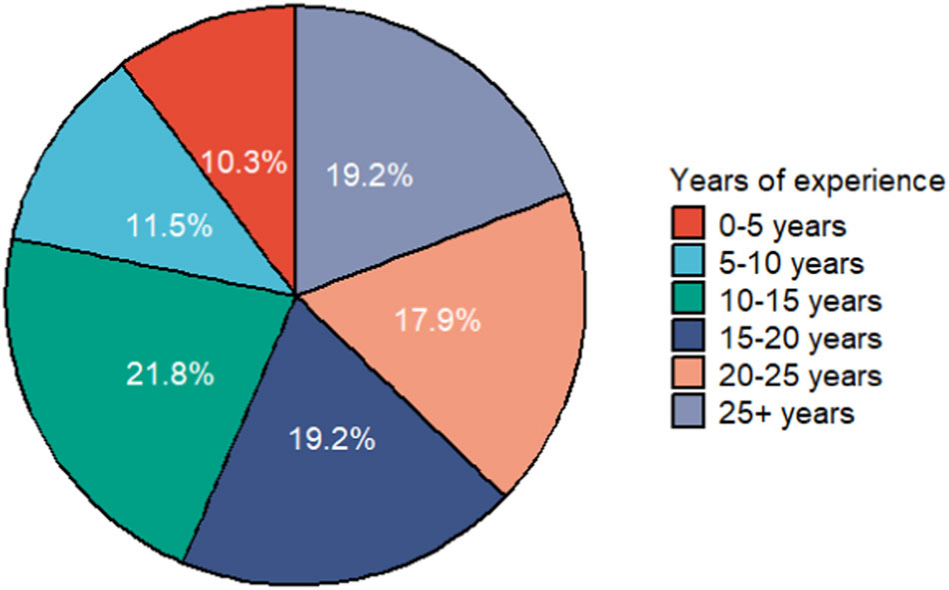
Years of clinical experience in multiple sclerosis

**Fig. 2:**
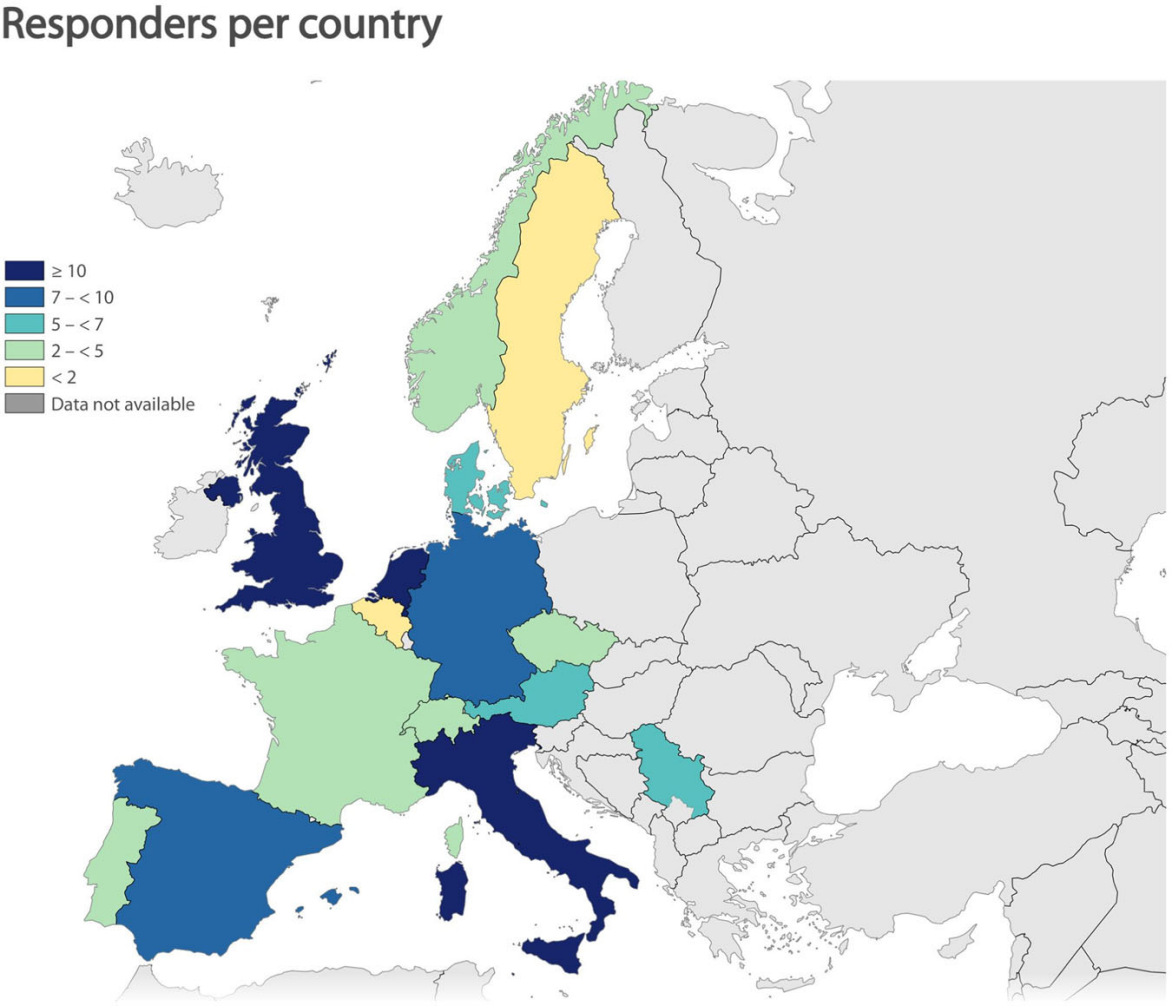
Map of the number of respondents per country. Countries that are shaded gray had no responders

For the first questionnaire iteration, the response rate was (49.6%). The respondents comprised 36 neurologists, 38 (neuro)radiologists, 2 biomedical engineers, and 1 physicist. The years of clinical experience in MS of responders are detailed in Fig. 1. In total, 93.6% work in an academic hospital, 29.5% in a research center, 3.8% in a general hospital, 7.7% in a private hospital/clinic, and 28.2% in an MS center/clinic. Responders could select multiple answers regarding their current work environment. The responders originated from 15 European countries (see Fig. 2). Among these responders, 86% reported having specialized MS (neuro)radiologists and neurologists in their center, 7.7% had (neuro)radiologists only, and 5.1% had only specialized MS neurologists. The mean percentage of responders without “I don’t know” answers was 91.2% for the first questionnaire and 95.1% for the second abbreviated questionnaire.

#### General information

Consensus was achieved on the inclusion of the following information—highly desired: name, age, sex, patient ID, date of scan; desired: diagnosis, disease severity score, disease-modifying therapy (DMT) type and onset, and relapses since the last scan. There was also a clear trend of support for including information on disability progression independent of relapses since the last scan (44% of responders), but no consensus was reached. Next to patient information, the following exam information was highly desired: scanner type, field strength, and whether gadolinium was administered. Additionally, the basic sequence parameters, such as Echo Time, were desired. As reports may become exhaustive and not every measure may be of interest for each scan, responders agreed (69.2%) that there should be an option to select a basic (including only a few measures) or an advanced report. Regarding the generation of models using reference data to contextualize single-subject results, responders agreed that these models should ideally be adjusted for disease type, disease duration, and current medical history and medication.

#### Lesion measurements

An overview of which lesion measures were deemed most useful is provided in Table 1. In the clinical setting, stratification based on the following regional lesion locations was found to be useful for all lesion metrics: cortical and juxtacortical, periventricular, cerebellum, thalamus, brainstem, deep white matter, and optic nerve. These regions largely correspond to the McDonald criteria for dissemination in space in MS diagnosis. Additionally, it was highly desired (75.6%) to include a section on the fulfillment of the McDonald criteria. Also, 82.2% of responders agreed that information on the presence of the central vein sign (CVS) is useful. For spinal cord analysis, the spinal cord level (e.g., C2/C3) is highly desired and the axial description (lateral/anterior) is desired.

**Table 1:** Recommendations for lesion measurements in commercial quantitative reports for multiple sclerosis for the purpose of diagnosis, prognosis, and monitoring

Cross-sectional	Diagnosis	Prognosis	Monitoring
Brain			
Absolute T2 lesion count	**Highly desired**	**Desired**	N.A.
Absolute T2 lesion volume	**Desired**	**Highly desired**	N.A.
Relative T2 lesion count			
To an MS reference population	**Desired**	**Highly desired**	N.A.
To an HC reference population	**Desired**	Not	N.A.
Relative T2 lesion volume			
To an MS reference population	**Desired**	**Highly desired**	N.A.
To an HC reference population	**Desired**	Not	N.A.
Absolute T1 lesion count	Not	**Desired**	N.A.
Absolute T1 lesion volume	Not	**Desired**	N.A.
Absolute Gd-enhancing count	**Highly desired**	N.A.	**Highly desired**
Proportion of T2 lesions that show enhancement	**Desired**	N.A.	**Desired**
T2-FLAIR hypointense lesion count	Not	**Desired**	N.A.
T2-FLAIR hypointense lesion count	Not	**Highly desired**	N.A.
PRL count	**Desired**	**Desired**	N.A.
Spinal cord			
Focal lesion count	**Highly desired**	**Highly desired**	N.A.
Focal lesion count relative to an MS reference population	**Desired**	**Highly desired**	N.A.
Gd-enhancing lesion count	**Highly desired**	**Desired**	N.A.
Proportion of T2 lesions that show enhancement	**Desired**	**Desired**	N.A.
Optic nerve			
Lesion count	**Highly desired**	**Desired**	N.A.
Lesion length	**Desired**	Not	N.A.
Lesion laterality	**Desired**	Not	N.A.
Lesion location	**Highly desired**	Not	N.A.
Longitudinal	Diagnosis	Prognosis	Monitoring
Brain			
Absolute T2 lesion count (new)	**Desired**	**Desired**	**Highly desired**
Absolute T2 lesion volume (change)	Not	**Desired**	**Highly desired**
Absolute T1 lesion count (new)	Not	**Desired**	**Desired**
Absolute T1 lesion volume (change)	Not	**Desired**	**Desired**
Absolute Gd-enhancing lesion count (new)	**Desired**	**Desired**	**Highly desired**
Absolute Gd-enhancing lesion volume (change)	Not	Not	**Desired**
PRL count (new)	Not	**Desired**	**Highly desired**
Spinal cord			
Focal lesion count (new)	**Desired**	**Desired**	**Highly desired**
Gd-enhancing lesion count (new)	**Desired**	**Highly desired**	**Highly desired**
Proportion of T2 lesions that show enhancement (new)	Not	Not	**Desired**
Optic nerve			
Lesion count (new)	Not	Not	**Desired**
Lesion length (increase)	Not	Not	**Desired**
New lesion location	Not	Not	**Highly desired**

Highly desired ≥ 75% agreement, desired ≥ 50% < 75% agreement

*MS* multiple sclerosis, *HC* healthy controls, *Gd* gadolinium, *FLAIR* fluid attenuated inversion recovery, *PRL* paramagnetic rim lesion

#### Volume and area measurements

The desired brain volume structures to be included in cross-sectional and longitudinal QReport analyses are detailed in Table 2. Classification of cortical measurements into lobes was not desired. The responders reached a consensus that in a cross-sectional setting, regional brain volumes should be included in the report as a percentage of the intracranial volume and contextualized with healthy and MS reference data.

**Table 2:** Recommendations for cross-sectional and longitudinal volume measurements for commercial quantitative reports in multiple sclerosis for the purposes of prognosis and monitoring

Structure	Prognosis	Monitoring
Total brain volume	**Highly desired**	**Highly desired**
Total GM volume	**Highly desired**	**Highly desired**
Cerebral GM volume	**Desired**	**Highly desired**
Cerebellar GM volume	Not	Not
Cortical thickness	**Desired**	**Desired**
Total CSF volume	Not	Not
Cerebral cortex volume	**Desired**	**Desired**
Cerebellar cortex volume	Not	Not
Total WM volume	**Desired**	**Highly desired**
Cerebral WM volume	**Desired**	**Desired**
Cerebellar WM volume	Not	**Desired**
Total ventricular volume	**Desired**	**Desired**
Thalamic volume	**Desired**	**Desired**
Basal ganglia volume	Not	Not
Hippocampus volume	Not	Not
Amygdala volume	Not	Not
Nucleus accumbens volume	Not	Not
Brainstem volume	**Desired**	**Desired**
Corpus callosum volume	**Desired**	**Desired**
Mesencephalon volume	Not	Not
Pons	Not	Not
Medulla oblongata	Not	Not
Hypothalamus	Not	Not
Lateral ventricles	Not	Not
Third ventricle	Not	Not
Fourth ventricle	Not	Not
Precuneus	Not	Not
Spinal cord		
Cervical cord volume	**Desired**	Not
Total cord volume	**Desired**	Not

Highly desired ≥ 75% agreement, desired ≥ 50% < 75% agreement

*MS* multiple sclerosis, *HC* healthy controls, *GM* gray matter, *WM* white matter

The consensus was reached that longitudinal regional brain volume is most useful as a percentile contextualized by healthy data and as atrophy rates. Also, responders agreed that atrophy rates would be useful when contextualized with both MS and healthy reference data. A total of 46% agreed that absolute volume change should be included. 64.7% supported the inclusion of changes in global cortical thickness. Changes in regional cortical thickness were not desired, although favored by 40% of respondents. It was desired (67.9%) to provide clinicians with an option to manually select the scans (or time points) to be included in the longitudinal QReport. Next to volume measurements, cross-sectional mean upper cervical cord area (MUCCA) is highly desired for prognosis purposes and desired for monitoring purposes.

### Visualization

It was considered desirable by the majority (68%) to visualize lesion segmentation outputs, allowing clinicians to switch between turning overlays and/or contours off and on. Furthermore, there was agreement that companies should include both lesion outlines and overlays with location-specific colors (e.g., periventricular lesion = red, juxtacortical lesions = blue, etc.). For brain segmentation, a visualization similar to that for lesion visualization was desired; responders agreed that brain segmentation overlays and outlines should be included, with a function to toggle them on and off.

### Clinical workflow

The significant majority (89.4%) deemed it desirable to use the QReport for every MS patient. Responders indicated that the time investment during the clinical workflow should not exceed 5 min (62.8%), with 20.5% desiring the QReport software to run completely automatically, without user intervention. QReports were deemed most beneficial for patients on DMT when used longitudinally and for monitoring purposes. The quality of the QReports should be assessed by (neuro)radiologists with the additional help of neurologists or a dedicated technician. 56.4% of the respondents agreed that both the radiologists and neurologists share responsibility for interpreting the results of the QReport, but only after approval of the QReport by the (neuro)radiologists (74.4%). It was deemed highly desirable (90.1%) that the lesion and atrophy quantification pipeline should be fully automated. There was no consensus for automation of quality control. 71.8% agreed that every requested clinical MRI scan containing the MS protocol should be processed fully automatically. It was desired that the use of QReports should be included in the standard reimbursement costs. Regarding patient access to QReports: 68.5% are in favor, thereof 24.5% suggested with constraints, but these constraints were not explicitly asked. It was desired to implement a simplified patient-oriented QReport.

### Clinical usefulness

Responders agreed that QReports would be used for diagnosis (71.4%), prognosis (84.4%), monitoring (96.1%), and treatment decisions (67.5%). There was a consensus that automated lesion segmentation aids radiological reporting (48.7% of the responders agreed and another 35.9% strongly agreed). Moreover, there was a consensus that QReports can improve the quality of care in MS (46.2% agreed and 39.7% strongly agreed).

### Deployment procedure

The vast majority (98.2%) deemed it highly desirable that commercial QReport implementations should consider two different formats. These answers were based on occupational preferences. Radiologists indicate a preference for a DICOM encapsulated PDF format in their work environment, while neurologists lean towards viewing the QReport in the electronic health record system. The preferred deployment procedure was mixed: 59.9% preferred cloud-based deployment, 37.5% preferred local hardware, and 30.3% preferred local virtualization. However, a large group of clinicians (28%) did not indicate any preference, in which case software can be deployed locally with center-specific IT. The preferred review system of the segmentations showcases distinct inclinations among responders: 50.0% prefer Picture Archiving and Communication System (PACS), 16.6% favor software-dedicated tools, and 54.2% are open to either option.

### Scanners and sequences

Most responders (75.8%) were willing to acquire a 3D-T1-weighted (T1w) scan next to the routinely acquired 3D-fluid attenuated inversion recovery (FLAIR), despite the additional scanning time and costs. It was, however, considered desirable (60%) that commercial QReports that only require 3D-FLAIR should be developed. The following scanner vendors were used by responders in normal clinical routine: GE (32.2%), Siemens (67.9%), and Philips (50.0%), while other vendors, though inquired about, were not utilized.

### Validation and testing

The adoption of quantitative MRI metrics in clinical practice is contingent upon validation. In total, 65% of responders seek some form of validation before using the QReport in daily clinical practice. 26% of responders reported that they are already convinced of the value and validity of QReports. It was deemed desirable by 65% that QReports need to be extensively validated before making it into daily clinical care. Responders’ opinions about the type of validation necessary varied: 70.6% emphasize multicenter clinical validation, 32.0% prefer randomized clinical trials, 45.3% suggest in-house clinical validation, and 48.0% advocate for in-house technical validation, again multiple options were possible. According to questionnaire results, barriers hindering the implementation of QReports in clinical practice were cost (63.5%), reliability (55.4%), and accessibility (55.4%).

### Quality control and confidence

Inclusion of levels of confidence and uncertainty in QReports is highly desired (83.8%). However, 12.2% expressed difficulty in incorporating such measures. Manual quality control that allows editing of the segmentation and volumetric results in the processing pipeline is favored by 69.7% of responders. 19.7% believed segmentation and volumetric results should not be editable. It was desired that a measurement can be rejected based on the radiologist’s judgment of the segmentation quality, while other measurements can still be included in the QReport. Preferences regarding quality control measures in QReports varied; 61.5% would include signal-to-noise ratio (SNR) and contrast-to-noise ratio (CNR) as measures of image quality, model fit measures, and artifact detection. 26.2% would include only SNR, 18.5% only CNR, 38.5% only artifact detection, and 26.2% only measures regarding model fit.

### QReport landscape

72.4% of responders indicated that they do not currently have implemented commercial QReports. This includes direct negatives (e.g., “No,” “No commercial QReport has been implemented”) and instances where only non-commercial methods are used (e.g., “only testing informally,” “not routinely implemented”). The main reasons for this were: (1) Cost concerns: 27.3% of responses mentioned cost as a significant issue, including the need for ongoing payments after initial free use and general affordability. (2) Integration challenges: 25% mentioned difficulties with integration into existing systems such as PACS, emphasizing the need for seamless integration. (3) Time constraints: 22.7% cited the time required to generate reports or integrate the tool as a bottleneck that is not compatible with clinical practice. (4) Data protection concerns: 15.9% raised issues related to data security and privacy, with some responders expressing distrust towards external QReport providers. (5) Reliability, quality, and validity issues: 15.9% reported concerns about the reliability of the reports, particularly in multicenter settings and with various scanner types, as well as overall trust in AI results following early failures when internally tested. (6) Interpretation and training needs: 13.6% found difficulties in understanding how to interpret QReports and how measurements were made, indicating a need for better training and clarity in report outputs. Technical and administrative barriers were mentioned, such as the lack of lesion maps, insufficient automation, and administrative resistance to adopting new technologies. Other less frequently mentioned issues included the physical and logistic constraints of requiring separate login terminals and the compatibility with local imaging interfaces.

## Discussion

The increasing use of MRI for diagnosis and monitoring of MS and increased sophistication of image analysis and quantification of damage have sparked the development of novel QReports to include the extent and severity of inflammatory and neurodegenerative aspects of the disease in clinical routine. This study identified the user requirements for and barriers hindering the adoption of QReports for the diagnosis and management of MS. The use of a Delphi panel method facilitated a structured and systematic consensus on key aspects of QReport development and application in clinical practice by clinicians with expertise in MS. Strong consensus was reached on the importance of including detailed lesion information and specific brain and spinal cord volume measurements in QReports. However, concerns were raised about the impact on report generation time, data protection, and the reliability of automated analysis. Below, we discuss each area of consensus, identify challenges, and provide recommendations to improve QReports adoption in clinical practice.

### Report measurements

Key metrics identified by the panel included absolute T2 lesion count and volume, which are highly desired for diagnosis and prognosis, respectively, aligning with literature that emphasizes the significance of lesion count in diagnosing MS and understanding its progression [10]. Relative T2 lesion count and volume, compared to both MS and healthy control reference populations, are also highlighted as desirable, illustrating the value of benchmarking against normative data. The absolute gadolinium-enhancing lesion count is highly desired for both diagnosis and monitoring, reinforcing its importance in detecting active inflammation [2]. Next to lesion measurements, key brain volume measurements included total brain volume and GM volume, which were highly desired for both monitoring and prognosis purposes. Brain atrophy has been shown to be a better predictor for clinical progression than lesion measures [11]. Unexpectedly, the absolute new T2 lesion count and the absolute new Gd-enhancing lesion count, despite their importance for demonstrating dissemination in time for diagnosis, were marked as “Desired” rather than “Highly desired.” This could be attributed to some respondents misunderstanding the question. For instance, one respondent explicitly questioned how “new” lesions could be determined at diagnosis, interpreting the term “diagnosis” as referring only to the first scan without prior imaging for comparison. This misunderstanding may have led to the underestimation of these metrics’ importance in the context of QReports.

Based on the survey results, we recommend that parties developing QReports in MS at least include the measures that are highly desired, as detailed in Tables 1 and 2. Currently, several companies have developed regulatory-approved lesion segmentation and atrophy quantification tools [7]. However, as indicated in a recent review and by responses to the questionnaire included in this study, lesion segmentation and detection tools have been integrated to a limited extent into clinical workflow [12]. It must be noted that the survey was designed to capture the ideal preferences and priorities of clinicians, focusing on their “wish list” instead of the current state of technical feasibility. While several of the requested features, such as lesion count, lesion volume, and brain volume measurements, are already technically feasible and—to a variable degree—incorporated into FDA and EMA-approved software solutions, some of the report measurements suggested here, such as spinal cord measurements, are still in their very early stages. Other preferences such as the inclusion of paramagnetic rim lesion count and the central vein sign presence, require additional sequences (e.g., susceptibility-weighted imaging) to be added to the current clinical protocol. Furthermore, several measures, such as brain volumes as percentage of the ICV, need careful consideration as people with bigger brains have more brain reserve [13].

Most responders (85.9%) indicated that they believe QReports can improve the quality of care in MS, which is also supported by two validation studies [8, 14]. However, a large group (72.4%) currently do not implement QReports in their clinical work, highlighting the remaining challenges preventing clinical implementation.

## Challenges

### Integration into clinical workflow

A primary concern expressed by clinicians was barriers to the integration of QReports into existing clinical workflows. While automated lesion segmentation and volumetric analysis hold the promise of enhancing diagnostic accuracy and monitoring disease progression, the actual integration process can be cumbersome. Issues such as the time required to process and review reports and difficulties in seamlessly merging these tools with hospital information systems like PACS and electronic health records, significantly impact their practical use. Embedding QReports directly into PACS or EHR systems using plug-ins or APIs can mitigate these issues, allowing clinicians to access reports alongside imaging data without switching platforms. Additionally, automated processing pipelines, where imaging data is automatically analyzed and QReports are delivered in real-time, could reduce delays and minimize workflow disruption. To ensure ongoing quality and relevance of the QReports in clinical workflow, regular review and updates to methods should be assigned to a designated team or role. Our findings suggest a need for solutions that can be more easily adopted into the daily routines of clinical environments, possibly through enhanced software compatibility and streamlined operations that do not detract from clinical efficiency.

### Cost and accessibility

The cost of implementing and maintaining QReports was another significant barrier identified. Given the ongoing financial constraints in healthcare systems, the perceived high costs associated with these tools deter institutions from adopting them. There is a call for products that accommodate financial reality, which is not abundant in most public sector healthcare. Lowering these costs could make these technologies more accessible and widely used. Next to lowering costs, companies developing QReports could work closely with financial decision-making bodies, such as hospital boards and insurers, to demonstrate the tangible benefits of these tools. For example, studies could quantify the time savings for (neuro)radiologists through faster, more precise reporting enabled by QReports. This time efficiency could lead to increased productivity in radiology departments and reduced workload for clinicians, translating into potential cost savings for healthcare institutions. For example, a study showed that in mammography screening there was a 44% reduction in workload when using AI-assisted screening, with a similar cancer detection rate to standard screening [15].

Additionally, there is potential for QReports to improve patient outcomes by providing more detailed and actionable information for treatment decisions. This could lead to better treatment optimization, fewer complications, and potentially improved quality of life for patients. To strengthen this argument, further research is needed to demonstrate how the use of QReports impacts treatment decisions and long-term patient outcomes. Such studies could provide insights into whether QReports are cost-effective and if they improve overall benefits for patients and healthcare systems.

### Interpretability

The ability to correctly interpret and trust the output of QReports is crucial for their acceptance. This study pointed out that there is a substantial need for educational initiatives to enhance clinicians’ understanding of how these reports are generated and their relevance in clinical decision-making. Additionally, improving the transparency of the algorithms used and the data processed would likely enhance trust and reliance on these systems. However, an important limitation that warrants further exploration is the potential for over-reliance on automation. As QReports become more integrated into clinical workflows, there is a risk that clinicians may defer too heavily to algorithmic outputs, potentially overlooking errors, biases, or contextual factors that require human judgment. This over-reliance could undermine the critical role of clinical expertise in decision-making and lead to unintended consequences [16]. Addressing this challenge necessitates not only improving the transparency and reliability of QReports but also fostering a balanced approach in their use—where automation supports, rather than replaces, the clinician’s judgment. Future research should investigate strategies to mitigate this risk, ensuring that clinicians remain both informed and critically engaged with the tools they use.

### Validation and standardization

Responders emphasized the importance of rigorous validation and standardization across different clinical settings and equipment. The effectiveness and reliability of QReports can be compromised by variability in MRI scanners and protocols, making multicenter clinical validation and in-house technical validation crucial. For example, image harmonization or local adaptation strategies could be employed to account for this variability [17, 18]. To ensure effective and reliable results, QReports should follow the quantitative neuroradiology initiative framework [19], which provides a structured approach for implementation. This study adds to the framework by identifying clinician-driven requirements for QReport development and integration. A recent review showed that little effort has been made towards previously integrating MS lesion segmentation methods [12]. Standardizing these processes and ensuring consistent performance across diverse settings would improve clinician confidence in these tools. In addition, for some proposed QReport measurements, it is vital to establish clear cut-off values and further define their clinical significance to ensure they are not only accurate but also actionable in clinical practice [20]. To systematically address these challenges, the scientific community should focus on the following:

1. **Develop and validate clinically meaningful cut-off values**: Establish clear, evidence-based (normative) cut-off values for key QReport measurements, ensuring they have well-defined clinical relevance across different patient populations and clinical scenarios.
2. **Multicenter clinical validation**: Conduct multicenter studies to validate QReport tools across diverse clinical settings, ensuring they are robust against variability in patient population, MRI scanners, and imaging protocols.
3. **In-house technical validation**: Implement routine in-house technical validation procedures to ensure consistent performance of QReports within individual clinical environments.

### Data protection and security

Data protection emerged as another concern, especially given the sensitivity of medical data and the increasing regulations surrounding data privacy. Ensuring that these tools comply with local and international data protection standards is essential to protect patient information and institutional integrity, which in turn, could support broader implementation. To address these concerns, several actionable options can be considered:

1. **Adopt robust encryption methods:** Implement advanced encryption techniques for data storage and transmission to safeguard sensitive patient information against unauthorized access. This is already present for most electronic hospital environments and developers of QReports could make use of this expertise.
2. **Conduct regular compliance audits:** Establish routine assessments to ensure adherence to local and international data privacy regulations, such as the GDPR, or other region-specific standards.
3. **Incorporate role-based access controls:** Limit access to QReport data and tools based on user roles, ensuring that only authorized personnel can view or modify sensitive information.
4. **Provide user education on data privacy:** Train clinicians and technical staff on best practices for data handling, emphasizing their roles in maintaining data security within the QReport ecosystem.

## Limitations

This survey was not without limitations. First, the initial questionnaire was distributed through the MAGNIMS email network. Despite encouragement to distribute this questionnaire among fellow neurologists and (neuro)radiologists, the majority of responders were within this network. Second, the vast majority of responders work in academic hospitals with specialized MS clinicians present. This will under-represent non-academic clinicians. Third, the responders were primarily from European academic centers, introducing a bias that may not fully reflect the needs of the global market or community-based settings. Since QReport software targets a worldwide audience, future studies should include diverse participants to better capture international and non-academic perspective. Fourth, not every question was answered by the same number of responders. Exclusion of the “I don’t know” option could have led to an overestimation of consensus. This is especially true for the second questionnaire, where the response rate was generally lower. However, we observed a low fraction (< 9%) with “I don’t know” answers. Another limitation is the lack of detailed information on the familiarity and experience of respondents with QReport tools. Our findings reflect the current low adoption rates of these tools, which may, in part, reflect dissatisfaction with available products—a gap we aim to address. Future research should include measures of user familiarity with these technologies to provide a more comprehensive understanding of the results and inform the development of targeted solutions for varying levels of expertise.

## Conclusion

In conclusion, while QReports offer significant potential to improve the management of MS, their widespread adoption depends on addressing the financial, educational, and technical challenges identified in this study. Tailoring these tools to meet the specific needs of clinicians and seamlessly integrating them into clinical workflows has the potential to improve patient care and disease management in MS. The user requirements identified in this study could therefore represent an important step towards improving the utility and functionality of QReports.

## Supplementary information

ELECTRONIC SUPPLEMENTARY MATERIAL

## Abbreviations

CNR: Contrast-to-noise ratio
DMT: Disease-modifying therapy
FLAIR: Fluid attenuated inversion recovery
MRI: Magnetic resonance imaging
MS: Multiple sclerosis
PACS: Picture Archiving and Communication System
QReports: Quantitative radiological reports
SNR: Signal-to-noise ratio
