# Supplementary material for: User requirements for quantitative radiological reports in multiple sclerosis
Source: Eur Radiol. 2025 Apr 16;35(10):5967–78. doi: 10.1007/s00330-025-11544-x (PMC12417231; doi:10.1007/s00330-025-11544-x)
Supplement: Supplementary file 1 — ELECTRONIC SUPPLEMENTARY MATERIAL [file 330_2025_11544_MOESM1_ESM.pdf]

User requirements for quantitative radiological reports in multiple sclerosis

ELECTRONIC SUPPLEMENTARY MATERIAL

Supplementary table 1. Percentage of responses per question and percentage “I don’t know” answers for the first questionnaire.

Question	Number of I don’t know answers	Percentage of responders, without I don’t know
6	1	98.7%
7	3	96.2%
8	Lesion count: 0<br>Lesion volume: 3<br>Relative count to HC: 14<br>Relative count to MS: 10<br>Relative volume to HC: 15<br>Relative volume to MS: 13	Lesion count: 100%<br>Lesion volume: 96.2%<br>Relative count to HC: 82%<br>Relative count to MS: 87.2%<br>Relative volume to HC: 80.8%<br>Relative volume to MS: 83.3%
9	0	100%
10	Lesion count: 4<br>Lesion volume: 7<br>Relative count to MS: 16<br>Relative volume to MS: 15	Lesion count: 94.9%<br>Lesion volume: 91.0%<br>Relative count to MS: 79.5%<br>Relative volume to MS: 80.8%
11	6	92.3%
12	Lesion count: 0<br>Lesion volume: 10<br>Proportion of enhancing T2 lesions: 6	Lesion count: 100%<br>Lesion volume: 87.2%<br>Proportion of enhancing T2 lesions: 92.3%
13	4	94.9%
14	Lesion count: 15<br>Lesion volume: 17	Lesion count: 80.8%<br>Lesion volume: 78.2%
15	16	79.5%
16	New lesion count: 0<br>Volume change: 4<br>Relative new lesion count to MS: 28<br>Relative new lesion count to HC: 42<br>Relative new lesion count to MS: 30	New lesion count: 100%<br>Volume change: 94.9%<br>Relative new lesion count to MS: 64%<br>Relative new lesion count to HC: 46.2%<br>Relative new lesion count to MS: 61.5%

	Relative new lesion count to HC: 43	Relative new lesion count to HC: 44.9%
17	1	98.3%
18	New lesion count: 13<br>Volume change: 15<br>Relative new lesion count to MS: 35<br>Relative new lesion count to MS: 36	New lesion count: 83.3%<br>Volume change: 80.8%<br>Relative new lesion count to MS: 55.1%<br>Relative new lesion count to MS: 53.8%
19	6	92.3%
20	New lesion count: 4<br>Volume change: 29<br>Proportion of enhancing: 8	New lesion count: 94.9%<br>Volume change: 62.8%<br>Proportion of enhancing: 90.8%
21	5	93.6%
22	New lesion count: 27<br>Volume change: 29	New lesion count: 69%<br>Volume change: 66.7%
23	16	79.5%
24	N/A	N/A
25	TBv: 3<br>TGMv: 6<br>CGMv: 9<br>CerebellarGMv: 16<br>Cortical thickness: 11<br>tCSFv: 14<br>Cerebral cortex: 12<br>Cerebellar cortex: 19<br>Insular lobe: 18<br>Temporal lobe: 16<br>Occipital lobe: 17<br>Frontal lobe: 18<br>Parietal lobe: 18<br>Limbic lobe: 17<br>Cingulate lobe: 17	TBv: 96.2%<br>TGMv: 92.3%<br>CGMv: 88.5%<br>CerebellarGMv: 79.5%<br>Cortical thickness: 85.9%<br>tCSFv: 82.1%<br>Cerebral cortex: 84.6%<br>Cerebellar cortex: 75.6%<br>Insular lobe: 76.9%<br>Temporal lobe: 79.5%<br>Occipital lobe: 78.2<br>Frontal lobe: 76.9%<br>Parietal lobe: 76.9%<br>Limbic lobe: 78.2%<br>Cingulate lobe: 78.2%
26	tWMv: 4<br>Cerebral WMv: 7<br>Cerebellar WMv: 19<br>tVentricularv: 9<br>Thalamus: 12<br>Basal ganglia: 17<br>Hippocampus: 16<br>Amygdala: 17<br>Ncl. accumbens: 18<br>Brainstem: 17	tWMv: 94.9%<br>Cerebral WMv: 91.0%<br>Cerebellar WMv: 75.6%<br>tVentricularv: 88.5%<br>Thalamus: 84.6%<br>Basal ganglia: 78.2%<br>Hippocampus: 79.5%<br>Amygdala: 78.2%<br>Ncl. accumbens: 76.9%<br>Brainstem: 78.2%

	Corpus callosum: 17<br>Mesencephalon: 17<br>Pons: 15<br>Medulla oblongata: 15<br>Hypothalamus: 23<br>Lateral ventricle: 17<br>Third ventricle: 17<br>Fourth ventricle: 18<br>Precuneus: 22	Corpus callosum: 78.2%<br>Mesencephalon: 78.2%<br>Pons: 80.8%<br>Medulla oblongata: 80.8%<br>Hypothalamus: 70.5%<br>Lateral ventricle: 78.2%<br>Third ventricle: 78.2%<br>Fourth ventricle: 76.9%<br>Precuneus: 71.8%
27	12	84.6%
28	N/A	N/A
29	3	96.2%
30	4	94.9%
31	10	97.2%
32	1	98.7%
33	1	98.7%
34	1	98.7%
35	8	89.7%
36	3	96.2%
37	4	94.8%
38	1	98.7%
39	2	97.4%
40	2	97.4%
41	2	97.4%
42	N/A	N/A
43	0	100%
44	0	100%
45	4	94.8%
46	0	100%
47	0	100%
48	2	97.4%
49	22	71.8%
50	6	92.3%

51	8	89.7%
52	10	87.2%
53	16	79.5%
54	0	100%
55	4	94.8%
56	3	96.2%
57	4	94.8%
58	12	84.6%
59	7	91.0%
60	13	83.3%
61	5	93.6%
62	3	96.2%
63	Open question on what to include in future iterations of the questionnaire	N.A.

Supplementary table 2. Percentage of responses per question and percentage “I don’t know” answers for the 2nd abbreviated questionnaire.

Question	Number of I don’t know answers	Percentage of responders, without I don’t know
1	Relative count to MS: 1<br>Relative volume to MS: 2<br>Relative count to HC: 6<br>Relative volume to HC: 7	Relative count to MS: 98.4%<br>Relative volume to MS: 96.8%<br>Relative count to HC: 90.3%<br>Relative volume to HC: 88.7%
2	Lesion count: 5<br>Lesion volume: 6	Lesion count: 91.9%<br>Lesion volume: 90.3%
3	Lesion count: 9<br>Lesion volume: 9	Lesion count: 85.5%<br>Lesion volume: 85.5%
4	Count: 0<br>Relative count to MS: 1	Count: 100%<br>Relative count to MS: 98.4%
5	0	100%
6	Lesion count: 0<br>Proportion: 4	Lesion count: 100%<br>Proportion: 93.5%
8	Count: 2<br>Length: 1<br>Laterality: 6<br>Location: 3<br>Count (new): 2<br>Increase in length: 7<br>New lesion location: 2	Count: 96.8%<br>Length: 98.4%<br>Laterality: 90.3%<br>Location: 95.2%<br>Count (new): 96.8%<br>Increase in length: 88.7%<br>New lesion location: 96.8%
9	1	98.4%
10	Lesion count: 1<br>Lesion location: 4<br>New lesion count: 1<br>New lesion location: 3	Lesion count: 98.4%<br>Lesion location: 93.5%<br>New lesion count: 98.4%<br>New lesion location: 95.2%
11	0	100%
12	New lesion count: 1<br>Relative new lesion count to MS: 8	New lesion count: 98.4%<br>Relative new lesion count to MS: 87.1%
13	New lesion count: 0<br>New proportion with enhancement: 5	New lesion count: 100%<br>New proportion with enhancement: 91.9%
15	5	91.9%
16	0	100%

17	5	91.9%
18	N/A	N/A
19	N/A	N/A

Commercial atrophy and lesion measurements

Project aim: The development of commercial quantitative radiological reporting tools has rapidly increased in recent years. Several companies have developed tools for use in multiple sclerosis providing clinicians, and potentially patients, with automated analysis of MRI scans and quantification of brain and lesion volumes often contextualized with reference data. Although several tools for use in MS are available and on the market, the perspective of clinicians as end-users on how such tools should be developed for optimal clinical usefulness has not been systematically identified and synthesized. In this project, we will draft a white paper to establish a consensus of expert clinicians on the development, features and structure of automated quantitative reports (**QReports**) in MS. We aim to encourage companies to produce tools based on the requirements of the clinicians as end-users. To establish an expert consensus, we will use the Delphi panel method interviewing clinicians from several leading institutes in the field of MS.

Delphi panel method: We will collect all the questionnaires and **compile the comments and answers**. A copy of the compiled comments **and answers** is sent to each participant along with the opportunity to comment further. At the end of each comment session, all questionnaires are returned to the **authors**, who will decide if another round is necessary or if the results are ready for publishing.

The questionnaire rounds can be repeated as many times as necessary to achieve consensus.

The questionnaire consists of 63 questions and can be saved in between sessions. It takes about 25-30 minutes to complete the questionnaire. **Please keep in mind that all the questions are about clinical implementation of quantitative reporting tools and not research purposes.**

We will collect your email address solely for the purpose of potential follow-up rounds to achieve consensus. However, to ensure anonymity during the processing of responses, we will not include email addresses in any of our analysis or reports.

Please forward this link to any other colleagues that could participate.
Thank you very much for your time.

* Indicates required question

1. Email *

Information about participant

2. 1. What is your profession? *

Tick all that apply.

- [ ] Neurologist
- [ ] Radiologist
- [ ] Neuroradiologist
- [ ] Other: ____________________

3. 2. How many years of clinical experience in MS do you have? *

Mark only one oval.

- ( ) 0-5 years
- ( ) 5-10 years
- ( ) 10-15 years
- ( ) 15-20 years
- ( ) 20-25 years
- ( ) 25+ years

4. 3. What setting do you practice in? **multiple answers are possible.** *

Tick all that apply.

- [ ] Academic hospital
- [ ] Research setting
- [ ] General hospital
- [ ] Private hospital/clinic
- [ ] MS center/clinic
- [ ] Other: ____________________

5. 4. Are there specialized MS (neuro)radiologists and neurologists available in your clinic? *

Mark only one oval.

- Yes
- Only (neuro)radiologists
- Only neurologists
- No
- I don't know
- Other: ____________________

6. 5. In which country do you currently work the most? *

____

Report measurements

The following questions will be about which measurements should be included in the QReport. Please leave any additional comments about a specific question in the comment box at the end of the section and note the question number.

7. 6. Which patient information should be included in the QReport? Please keep in mind that not all options below are in the dicom header. **multiple answers are possible.** *

Tick all that apply.

- [ ] Name
- [ ] Age
- [ ] Sex
- [ ] Patient ID
- [ ] Date of scan(s)
- [ ] Date of diagnosis (if applicable)
- [ ] Disease severity score (EDSS) (if available)
- [ ] Disease modifying therapy + startdate (if applicable)
- [ ] Relapses since last scan (if available)
- [ ] Disability progression independent of relapse since last scan (if available)
- [ ] I don't know
- [ ] Other: ______________________

8. 7. Which scanner-related information should be included in the QReport? **multiple answers are possible.** *

Tick all that apply.

- [ ] Scanner type
- [ ] Field strength
- [ ] Type of receiver coil used
- [ ] Sequence parameters
- [ ] If Gadolinium was administered
- [ ] I don't know
- [ ] Other: ______________________

CROSS-SECTIONAL LESION MEASUREMENTS

The following questions are about the cross-sectional lesion measurements that should be included in the QReport.

Please leave any additional comments about a specific question in the comment box at the end of the section and note the question number.

9. 8. Should the following **cross-sectional T2-**lesion measurements be included in the QReport? If yes, for which purpose would you use it? Please select the pupose(s). **multiple answers per row are possible.** *

Tick all that apply.

	Diagnosis	Prognosis	Not relevant	I don't know
**Lesion count**	☐	☐	☐	☐
**Lesion volume**	☐	☐	☐	☐
**Relative lesion count contextualised with MS reference population**	☐	☐	☐	☐
**Relative lesion count contextualised with healthy reference population**	☐	☐	☐	☐
**Relative lesion volume contextualised with MS reference population**	☐	☐	☐	☐
**Relative lesion volume contextualised with healthy reference population**	☐	☐	☐	☐

10. 9. Please select which of the following cross-sectional T2-lesion locations would be of interest for the QReport. **multiple answers are possible.** *

Tick all that apply.

- [ ] Cortical / juxtacortical lesions
- [ ] Periventricular lesions
- [ ] Cerebellum lesions
- [ ] Brain stem lesions
- [ ] Deep white matter lesions
- [ ] Internal capsule
- [ ] Hippocampus lesions
- [ ] Thalamus lesions
- [ ] Corpus callosum lesions
- [ ] Spinal lesions
- [ ] None of the above
- [ ] I don't know
- [ ] Other: ____________________

11. 10. Should the following **cross-sectional T1** hypointense lesion measurements * be included in the QReport? If yes, for which purpose would you use it? Please select the purpose(s), **multiple answers per row are possible.**

Tick all that apply.

	Diagnosis	Prognosis	Not relevant	I don't know
**Lesion count**	☐	☐	☐	☐
**Lesion volume**	☐	☐	☐	☐
**Relative lesion count contextualised with MS reference population**	☐	☐	☐	☐
**Relative lesion volume contextualised with MS reference population**	☐	☐	☐	☐
**Relative lesion count contextualised with HC reference population**	☐	☐	☐	☐
**Relative lesion volume contextualised with HC reference population**	☐	☐	☐	☐

12. 11. Please select which of the following cross-sectional T1 hypointense lesion locations would be of interest for the QReport. **multiple answers are possible.** *

Tick all that apply.

- [ ] Cortical / juxtacortical lesions
- [ ] Periventricular lesions
- [ ] Cerebellum lesions
- [ ] Brain stem lesions
- [ ] Deep white matter lesions
- [ ] Internal capsule lesion
- [ ] Hippocampus lesions
- [ ] Thalamus lesions
- [ ] Corpus callosum lesions
- [ ] Spinal lesions
- [ ] None of the above
- [ ] I don't know
- [ ] Other: ____________________

13. 12. Should the following **cross-sectional Gd-enhancing** lesion measurements be included in the QReport? Please select the purpose(s), **multiple answers per row are possible.** *

Tick all that apply.

	Diagnosis	Monitoring	Not relevant	I don't know
**Lesion count**	☐	☐	☐	☐
**Lesion volume**	☐	☐	☐	☐
**Proportion of T2 lesions that show enhancement**	☐	☐	☐	☐

14. 13. Which of the following cross-sectional Gd-enhancing lesion locations would be of interest for the QReport? **multiple answers are possible.** *

Tick all that apply.

- [ ] Cortical /juxtacortical lesions
- [ ] Periventricular lesions
- [ ] Cerebellum lesions
- [ ] Brain stem lesions
- [ ] Deep white matter lesions
- [ ] Internal capsule lesions
- [ ] Hippocampus lesions
- [ ] Thalamus lesions
- [ ] Corpus callosum lesions
- [ ] Spinal lesions
- [ ] None of the above
- [ ] I don't know
- [ ] Other: ____________________

15. 14. Should the following **cross-sectional T2-FLAIR hypointense** lesion measurements be included in the QReport? Please select the purpose(s), **multiple answers per row are possible.** *

Tick all that apply.

	Diagnosis	Monitoring	Not relevant	I don't know
**Lesion count**	☐	☐	☐	☐
**Lesion volume**	☐	☐	☐	☐

16. 15. Which of the following cross-sectional T2-FLAIR hypointense lesion locations would be of interest for the QReport? **multiple answers are possible.** *

Tick all that apply.

- [ ] Cortical /juxtacortical lesions
- [ ] Periventricular lesions
- [ ] Cerebellum lesions
- [ ] Brain stem lesions
- [ ] Deep white matter lesions
- [ ] Internal capsule lesions
- [ ] Hippocampus lesions
- [ ] Thalamus lesions
- [ ] Corpus callosum lesions
- [ ] Spinal lesions
- [ ] None of the above
- [ ] I don't know
- [ ] Other: ______________________

LONGITUDINAL LESION MEASUREMENTS

The following questions are about the longitudinal lesion measurements that should be included in the QReport. Please leave any additional comments about a specific question in the comment box at the end of the section and note the question number.

17. 16. Should the following **longitudinal T2-**lesion measurements be included in the QReport? If yes, for which purpose would you use it? Please select the purpose(s), **multiple answers per row are possible.** *

Tick all that apply.

	Diagnosis	Monitoring	Prognosis	Not relevant	I don't know
**New lesion count**	☐	☐	☐	☐	☐
**Volume change**	☐	☐	☐	☐	☐
**Relative new lesion count contextualised with MS reference population**	☐	☐	☐	☐	☐
**Relative new lesion count contextualised with healthy reference population**	☐	☐	☐	☐	☐
**Relative volume change contextualised with MS reference population**	☐	☐	☐	☐	☐
**Relative volume change contextualised with healthy reference population**	☐	☐	☐	☐	☐

18. **17.** Which of the following **new** T2-lesion locations would be of interest for the QReport? **multiple answers are possible.** *

Tick all that apply.

- [ ] Cortical / juxtacortical lesions
- [ ] Periventricular lesions
- [ ] Cerebellum lesions
- [ ] Brain stem lesions
- [ ] Deep white matter lesions
- [ ] Internal capsule lesions
- [ ] Hippocampus lesions
- [ ] Thalamus lesions
- [ ] Corpus callosum lesions
- [ ] Spinal lesions
- [ ] None of the above
- [ ] I don't know
- [ ] Other: ______________________

19. 18. Should the following **longitudinal T1** hypointense lesion measurements be included in the QReport? Please select the purposes, **multiple answers per row are possible.** *

Tick all that apply.

	Diagnosis	Monitoring	Prognosis	Not relevant	I don't know
**New lesion count**	☐	☐	☐	☐	☐
**Volume change**	☐	☐	☐	☐	☐
**Relative new lesion count contextualised with MS reference population**	☐	☐	☐	☐	☐
**Relative volume change contextualised with MS reference population**	☐	☐	☐	☐	☐

20. **19.** Which of the following **new** T1-lesion locations would be of interest for the QReport? **multiple answers are possible.** *

Tick all that apply.

- [ ] Cortical /juxtacortical lesions
- [ ] Periventricular lesions
- [ ] Cerebellum lesions
- [ ] Brain stem lesions
- [ ] Deep white matter lesions
- [ ] Internal capsule lesions
- [ ] Hippocampus lesions
- [ ] Thalamus lesions
- [ ] Corpus callosum lesions
- [ ] Spinal lesions
- [ ] None of the above
- [ ] I don't know
- [ ] Other: ____________________

21. 20. Should the following **longitudinal Gd-enhancing** lesion measurements be included in the QReport? Please select the purpose(s), **multiple answers per row are possible.** *

Tick all that apply.

	Diagnosis	Monitoring	Prognosis	Not relevant	I don't know
**New lesion count**	☐	☐	☐	☐	☐
**Volume change**	☐	☐	☐	☐	☐
**Proportion of Gd-enhancing lesions with respect to total lesion count**	☐	☐	☐	☐	☐

22. 21. Which of the following **new** Gd-enhancing lesion locations would be of interest for the QReport? **multiple answers are possible.** *

Tick all that apply.

- [ ] Cortical /juxtacortical lesions
- [ ] Periventricular lesions
- [ ] Cerebellum lesions
- [ ] Brain stem lesions
- [ ] Deep white matter lesions
- [ ] Internal capsule lesions
- [ ] Hippocampus lesions
- [ ] Thalamus lesions
- [ ] Corpus callosum lesions
- [ ] Spinal lesions
- [ ] None of the above
- [ ] I don't know
- [ ] Other: ______________________

23. 22. Should the following **longitudinal** T2-FLAIR **hypointense** lesion measurements be included in the QReport? Please select the purpose(s), **multiple answers per row are possible.** *

Tick all that apply.

	Diagnosis	Monitoring	Prognosis	Not relevant	I don't know
**New lesion count**	☐	☐	☐	☐	☐
**Volume change**	☐	☐	☐	☐	☐

24. 23. Which of the following **new** T2-FLAIR hypointense lesion locations would be of interest for the QReport? **multiple answers are possible.** *

Tick all that apply.

- [ ] Cortical / juxtacortical lesions
- [ ] Deep white matter lesions
- [ ] Periventricular lesions
- [ ] Cerebellum lesions
- [ ] Brain stem lesions
- [ ] Internal capsule lesions
- [ ] Hippocampus lesions
- [ ] Thalamus lesions
- [ ] Corpus callosum lesions
- [ ] Spinal lesions
- [ ] None of the above
- [ ] I don't know
- [ ] Other: ______________________

25. 24. Do you have any comments about the report measurement section?

____

____

____

____

____

Brain volume measurements

Please leave any additional comments about a specific question in the comment box at the end of the section and note the question number.

26. 25. Which of the following volumes of the cortical brain structures would you like to see in a cross-sectional and longitudinal QReport in a clinical setting? **multiple answers per row are possible.** *

Tick all that apply.

	Prognosis	Monitoring	Not relevant	I don't know
**Total brain volume**	☐	☐	☐	☐
**Total gray matter volume**	☐	☐	☐	☐
**Cerebral gray matter volume**	☐	☐	☐	☐
**Cerebellar gray matter volume**	☐	☐	☐	☐
**Cortical thickness**	☐	☐	☐	☐
**Total cerebrospinal fluid volume**	☐	☐	☐	☐
**Cerebral cortex**	☐	☐	☐	☐
**Cerebellar cortex**	☐	☐	☐	☐
**Insular lobe**	☐	☐	☐	☐
**Temporal lobe**	☐	☐	☐	☐
**Occipital lobe**	☐	☐	☐	☐
**Frontal lobe**	☐	☐	☐	☐
**Parietal lobe**	☐	☐	☐	☐

**Limbic lobe**	☐	☐	☐	☐
**Cingulate lobe**	☐	☐	☐	☐

27. 26. Which of the following volumes of the subcortical brain structures would you like to see in a **cross-sectional** and/or **longitudinal** QReport in a clinical setting? **multiple answers per row are possible.** *

Tick all that apply.

	Prognosis	Monitoring	Not relevant	I don't know
**Total white matter volume**	☐	☐	☐	☐
**Cerebral white matter volume**	☐	☐	☐	☐
**Cerebellar white matter volume**	☐	☐	☐	☐
**Total ventricular volume**	☐	☐	☐	☐
**Thalamus**	☐	☐	☐	☐
**Basal ganglia**	☐	☐	☐	☐
**Hippocampus**	☐	☐	☐	☐
**Amygdala**	☐	☐	☐	☐
**Nucleus accumbens**	☐	☐	☐	☐
**Brain stem**	☐	☐	☐	☐
**Corpus callosum**	☐	☐	☐	☐
**Mesencephalon**	☐	☐	☐	☐
**Pons**	☐	☐	☐	☐
**Medulla oblongata**	☐	☐	☐	☐
**Hypothalamus**	☐	☐	☐	☐

Lateral

Lateral ventricle	☐	☐	☐	☐
Third ventricle	☐	☐	☐	☐
Fourth ventricle	☐	☐	☐	☐
Precuneus	☐	☐	☐	☐

28. 27. Should cortical measurements be classified into lobes?

Mark only one oval.

- Yes
- No
- I don't know
- Other: ____________________

29. 28. Are there any other regional volume measurements that should be included in the QReport? *

____

____

____

____

____

30. 29. How would you like to see the regional volumes presented in a **cross-sectional** QReport? **multiple answers are possible.** *

Tick all that apply.

- [ ] As abolute volume(s) [cc]
- [ ] As fraction of the intracranial volume(s) [%]
- [ ] With reference to a healthy population [percentile/Z-score]
- [ ] With reference to a MS population [percentile/Z-score]
- [ ] I don't know
- [ ] Other: ____________________

31. 30. How would you like to see the regional volumes presented in a **longitudinal** QReport? **multiple answers are possible.** *

Tick all that apply.

- [ ] As abolute volume(s) change [cc]
- [ ] As fraction of the intracranial volume(s) [%]
- [ ] With reference to a healthy population [percentile/Z-score]
- [ ] With reference to a MS population [percentile/Z-score]
- [ ] As atrophy rates [%/year]
- [ ] As atrophy rates compared to a healthy population [percentile/Z-score]
- [ ] As atrophy rates compared to a MS population [percentile/Z-score]
- [ ] I don't know
- [ ] Other: ____________________

32. 31. Should the report include cortical thickness measurements? **multiple answers are possible.** *

Tick all that apply.

- [ ] Yes, global cortical thickness
- [ ] Yes, regional cortical thickness
- [ ] No measurements of thickness
- [ ] I don't know
- [ ] Other: ____________________

33. 32. Which scans should be included for the longitudinal QReport? *

Mark only one oval.

- Only the current and the most recent previous scan
- Option to select the timepoints manually depending on the purpose of the QReport
- All scans should be reported
- I don't know
- Other: ______________________

34. 33. Should the QReport include a section on the fullfillment of McDonald criteria? *

Mark only one oval.

- Strongly agree
- Agree
- Neutral
- Disagree
- Strongly disagree
- I don't know
- Other: ______________________

35. 34. Would you like flexibility in the QReport content, i.e. would you like to choose and alter the QReport content case-by-case? **multiple answers are possible.** *

Tick all that apply.

- [ ] No, there should only be one standard QReport
- [ ] Yes, there should be an option to choose between a basic (just a few measures) and advanced (all the reported measures) QReport
- [ ] Yes, I would like to choose which content should appear in the QReport manually
- [ ] I don't know
- [ ] Other: ______________________

36. 35. When contextualizing results to a reference population, which clinical aspects/factors should be taken into account for the model? Please keep in mind that it is valid only if enough data is available to stratify into groups. **multiple answers are possible.** *

Tick all that apply.

- [ ] Disease type (phenotype e.g. "increased atrophy for a RRMS patient")
- [ ] Disease duration (e.g. "for this duration of the disease, the patient has increased lesion volume")
- [ ] Current medical history/medication
- [ ] Last objective relapse
- [ ] Recent disease activity
- [ ] Disability progression
- [ ] None of the above
- [ ] All of the above
- [ ] I don't know
- [ ] Other: ______________________

37. Do you have any additional comments about the report measurement section? Please note the question number.

____

____

____

____

____

QReport visualisation & clinical workflow

Please leave any additional comments about a specific question in the comment box at the end of the section and note the question number.

38. 36. Should the lesion segmentation be visualised? **multiple answers are possible.** *

Tick all that apply.

- [ ] Yes, as overlays
- [ ] Yes, as contours
- [ ] Yes, as overlays with lesion-location-specific colours (e.g. periventricular lesions=red, juxtacortical lesions=blue)
- [ ] Yes, as contours with lesion-location-specific colours (e.g. periventricular lesions=red, juxtacortical lesions=blue)
- [ ] Option to switch overlays/contours off and on again
- [ ] No
- [ ] I don't know
- [ ] Other: ______________________

39. 37. Should the brain segmentation be visualised? **multiple answers are possible.** *

Tick all that apply.

- [ ] Yes, as overlays with the option to select each region separately
- [ ] Yes, as contours with the option to select each region separately
- [ ] Yes, with the overlays for every region
- [ ] Yes, with the contours for every region
- [ ] No
- [ ] I don't know
- [ ] Other: ______________________

40. 38. In which clinical situations would you use commercially available QReports for lesion and atrophy measurements? **multiple answers are possible.** *

Tick all that apply.

- [ ] Diagnosis
- [ ] Prognosis
- [ ] Monitoring
- [ ] Treatment decision-making
- [ ] All of the above
- [ ] I don't know
- [ ] Other: ______________________

41. 39. Would automated lesion segmentation aid radiological reporting? *

Mark only one oval.

- ( ) Strongly agree
- ( ) Agree
- ( ) Neutral
- ( ) Disagree
- ( ) Strongly disagree
- ( ) I don't know
- ( ) Other: ______________________

42. 40. QReports can improve the quality of care in MS *

Mark only one oval.

- Strongly agree
- Agree
- Neutral
- Disagree
- Strongly disagree
- I don't know
- Other: ____________________

43. 41. How much time in your clinical workflow would you be willing to invest in a QReport (e.g. quality control and reading the QReport)?

Mark only one oval.

- No time (should run automatically)
- 1-5 minutes
- 5-10 minutes
- 10-15 minutes
- 15-30 minutes
- More than 30 minutes
- I don't know
- Other: ____________________

44. 42. Would you use a QReport for every MS patient? If not, please specify the type of patient who would most benefit from these measurements. *

45. 43. Who would be responsible for assessing the quality of the QReport? **multiple answers are possible.**

Tick all that apply.

- [ ] Radiologist only
- [ ] Radiologist and neurologist together
- [ ] Dedicated technician
- [ ] Technician and radiologist
- [ ] Technician, radiologist and neurologist
- [ ] Quality control should be entirely automatic
- [ ] I don't know
- [ ] Other: ______________________

46. 44. Who would be responsible for interpreting the results of the report?

Mark only one oval.

- ( ) Radiologist only
- ( ) Neurologist only
- ( ) Radiologist and neurologist together
- ( ) I don't know
- ( ) Other: ______________________

47. 45. Which components of the QReport workflow should be fully automated? * **multiple answers are possible.**

Tick all that apply.

- [ ] Lesion pipeline
- [ ] Atrophy pipeline
- [ ] Quality control
- [ ] All of the above
- [ ] I don't know
- [ ] Other: ______________________

48. 46. The QReport should only be available to the neurologist once the radiologist has approved the QReport *

Mark only one oval.

- Strongly agree
- Agree
- Neutral
- Disagree
- Strongly disagree
- I don't know
- Other: ______________________

49. 47. Please select the optimal QReport workflow from requesting the scans to reviewing the QReport. **multiple answers are possible.** *

Tick all that apply.

- [ ] MRI exam is processed when the requesting neurologist selected the option for a QReport
- [ ] Every requested clinical MS protocol will be processed automatically
- [ ] The QReport is created after a request from the patient
- [ ] QReport will be created when the radiologist requests it at the radiologist work station
- [ ] All of the above
- [ ] I don't know
- [ ] Other: ______________________

50. Do you have any additional comments for the visualisation & clinical workflow section? Please note the question number.

____
____
____
____
____

Deployment procedure and scanners & sequences

Please leave any additional comments about a specific question in the comment box at the end of the section and note the question number.

Deployment procedure

The following questions are about how the software should be implemented in the clinic.

51. 48. How would you like to view the QReport? **multiple answers are possible.** *

Tick all that apply.

- [ ] As a (dicom encapsulated) PDF file in the radiologist work environment
- [ ] In the electronic health record/EMR of the patient
- [ ] I don't know
- [ ] Other: ____________________

52. 49. What is your preferred QReport deployment procedure? **multiple answers are possible.** *

Tick all that apply.

- [ ] Cloud-based
- [ ] Local hardware
- [ ] Local virtualisation
- [ ] I don't know
- [ ] Other: ____________________

53. 50. Where would you like to review the segmentations? **multiple answers are possible.** *

Tick all that apply.

- [ ] PACS system
- [ ] Software dedicated to the tool (such as a chrome browser)
- [ ] Either PACS or dedicated software
- [ ] I don't know
- [ ] Other: ____________________

Scanner and sequences

54. 51. Many current quantitative volumetric QReports require both 3D FLAIR and 3D T1-weighted input. Would it be feasible to acquire both these isotropic sequences in clinical routine? *

Mark only one oval.

- Yes
- No, only 3D FLAIR
- No, only 3D FLAIR and 2D T1
- No, only 2D FLAIR and 2D T1
- No, only 2D FLAIR and 3D T1
- I don't know
- Other: ______________________

55. 52. Which scanner vendors do you use in normal clinical routine? **multiple answers are possible.** *

Tick all that apply.

- [ ] GE
- [ ] Siemens
- [ ] Philips
- [ ] Canon medical
- [ ] I don't know
- [ ] Other: ______________________

56. 53. How should the tool be financed to be used in clinical routine? **multiple answers are possible.** *

Tick all that apply.

- [ ] Included in the standard reimbursement costs
- [ ] Additional payment
- [ ] Hospital funded
- [ ] Government funded
- [ ] I don't know
- [ ] Other: ____________________

57. Do you have any additional comments for the Deployment and scanners & sequences section? Please note the question number.

____
____
____
____
____

Validating & testing, Quality control and patient involvement section.

Please leave any additional comments about a specific question in the comment box at the end of the section and note the question number.

Validation and testing

58. 54. What is needed to convince you to use quantitative MRI metrics for monitoring and/or diagnostic decision-making in daily clinical practice? *

____
____
____
____
____

59. 55. Which barrier hampers you currently to implement QReports in daily clinical practice? **multiple answers are possible.** *

Tick all that apply.

- [ ] Costs
- [ ] Not reliable enough
- [ ] Not accessible
- [ ] I don't know
- [ ] Other: ________________

60. 56. What validation would be needed before implementation of the method in the clinic? **multiple answers are possible.** *

Tick all that apply.

- [ ] Randomized clinical trial
- [ ] In-house clinical validation
- [ ] Multi-center technical validation
- [ ] In-house technical validation
- [ ] I don't know
- [ ] Other: ________________

Quality control and confidence

61. 57. Should the QReport include measurements of confidence and uncertainty of quantitative results? *

Mark only one oval.

- ( ) Yes
- ( ) No
- ( ) I don't know
- ( ) It is very difficult to provide certainty measurements
- ( ) Other: ________________

62. 58. If quality control is manual, should segmentation and volumetric results be editable? *

Mark only one oval.

- Yes
- No
- I don't know
- Other: ____________________

63. 59. What would you prefer to do when the quality of the segmentation and/or volumetry results is insufficient? *

Mark only one oval.

- Reject all the measurements
- Reject specific measurements based on your judgement
- Manually adjust the segmentation
- I don't know
- Other: ____________________

64. 60. Which quality control measures should be included in the QReport? **multiple answers are possible.** *

Tick all that apply.

- [ ] SNR
- [ ] CNR
- [ ] Artefact (e.g. movement or aliasing)
- [ ] Algorithm model fit
- [ ] All of the above
- [ ] I don't know
- [ ] Other: ____________________

Patient involvement

65. 61. Should the patient be able to see the QReport? *

Mark only one oval.

- Yes
- No
- I don't know
- Other: ____________________

66. 62. Would a separate patient-oriented QReport be helpful? If yes, how would this be different from the original report? *

67. Do you have any additional comments for the Validating & testing, Quality control and patient involvement section? Please note the question number.

68. 63. Which topic was not reviewed in this questionnaire and should be included in the next circulation? Or: do you have any additional comments? *

This content is neither created nor endorsed by Google.

Google Forms

Second abbreviated questionnaire on commercial atrophy and lesion measurements

Dear responder,

First of all many thanks for filling out the questionnaire on the user requirements of commercial atrophy and lesions measurements. We know it was long and extensive. Following the Delphi-panel method we have gathered the responses and have adjusted the questionnaire based on additional suggestions. We have formulated or adjusted 20 questions and the questionnaire should now take 3-5 minutes to complete.

Please use the same email-address as in the first questionnaire so we can match your answers with your previous. If you don't remember please contact David van Nederpelt at: d.vannederpelt@amsterdamumc.nl

* Indicates required question

1. Email *

Report measurements

The following questions will be about which measurements should be included in the QReport. Please leave any additional comments about a specific question in the comment box at the end of the section and note the question number.

LESION MEASUREMENTS, CLARIFICATIONS

The following questions are about the cross-sectional lesion measurements that should be included in the QReport. Some questions required a bit of clarification, we have included some additional information. Please leave any additional comments about a specific question in the comment box at the end of the section and note the question number.

Example of graph contextualising single-subject lesion volume results (ml) with MS and HC reference data. The black dot represents the single-subject results, which is contextualised by the blue and green reference curves.

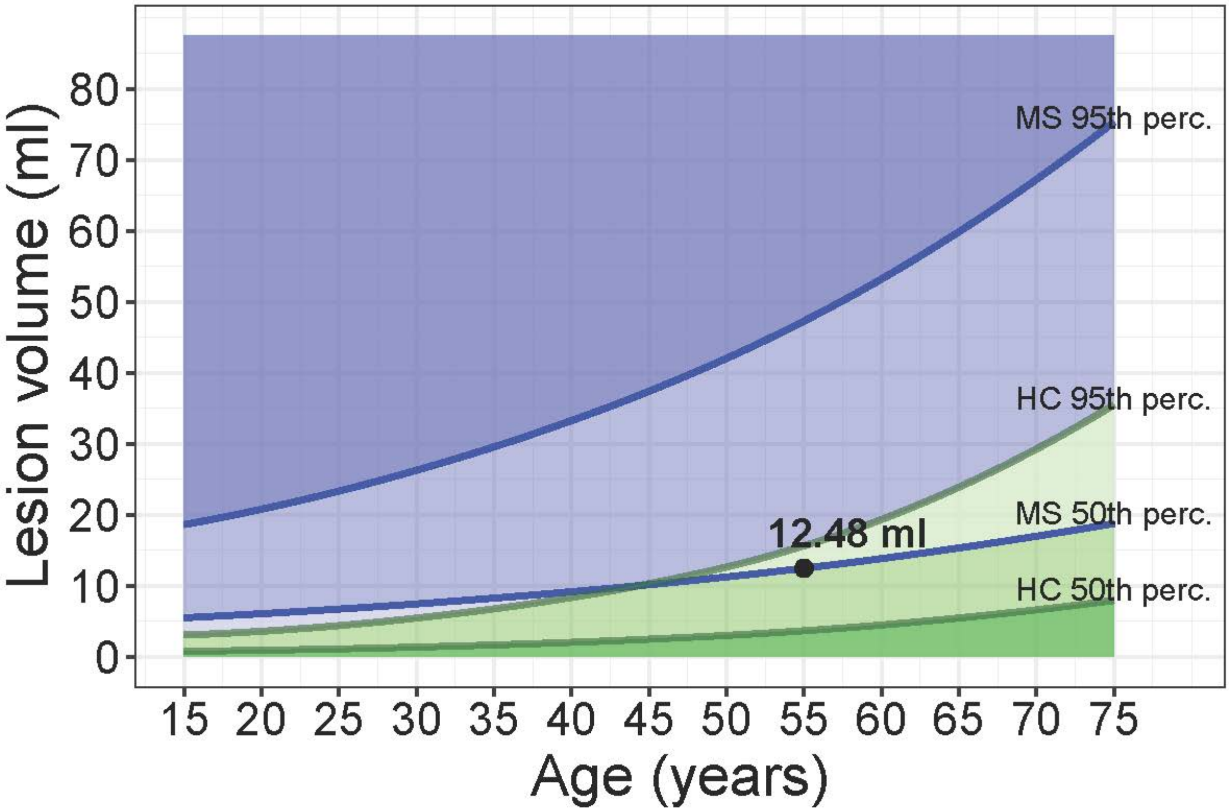

2. 1. In the previous questionnaire we included questions about absolute T2 lesion count and volume. For clarity, we have now added a figure (see above) showing an example of how reference data can be used to contextualise single-subject results. Please indicate if these measurements should be included in the QReport? Please select the purpose(s). **multiple answers per row are possible.** *

Tick all that apply.

	Diagnosis	Prognosis	Not relevant	I don't know
**Relative T2 lesion count (with reference to a MS reference population)**	☐	☐	☐	☐
**Relative T2 lesion volume (with reference to MS reference population)**	☐	☐	☐	☐
**Relative T2 lesion count (with reference to HC reference population)**	☐	☐	☐	☐
**Relative T2 lesion volume (with reference to HC reference population)**	☐	☐	☐	☐

Example of hypointense lesions (red circle) on FLAIR

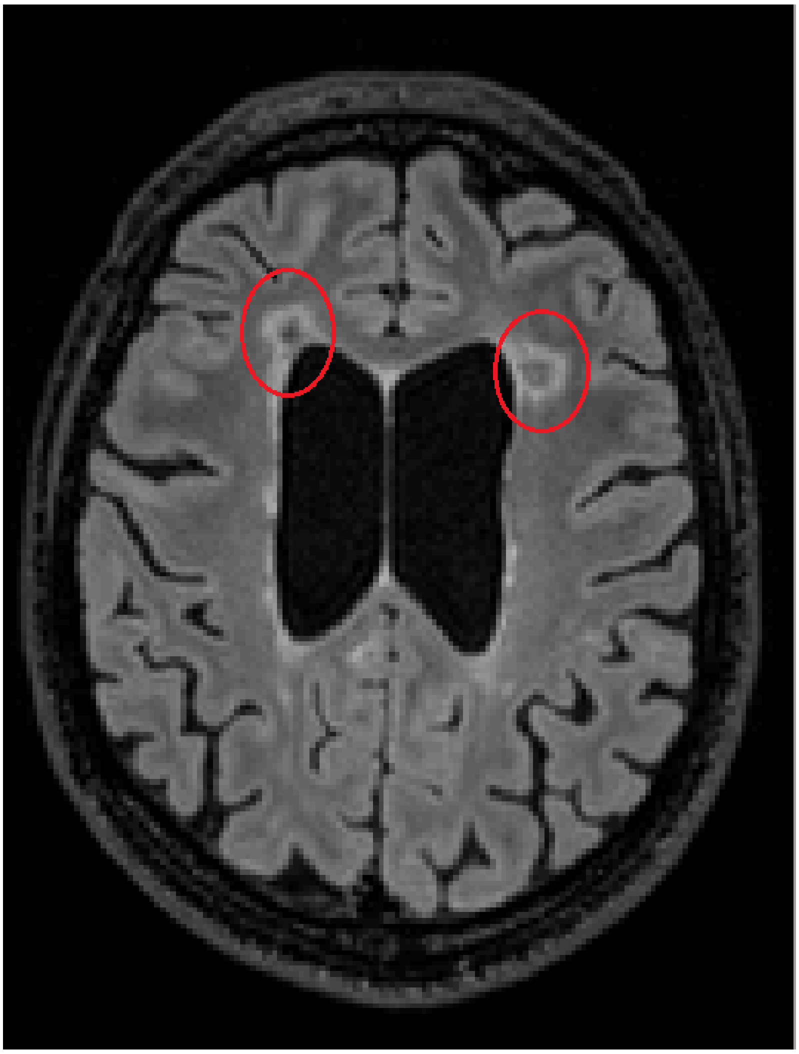

3. 2. In the previous questionnaire we included a question about **cross-sectional T2-FLAIR hypointense** lesion measurements. To increase clarity, we have added an example of such lesions. Please indicate if these measurements should be included in the QReport? Please select the purpose(s), **multiple answers per row are possible.** *

Tick all that apply.

	Diagnosis	Prognosis	Not relevant	I don't know
**T2-FLAIR hypointense lesion count**	☐	☐	☐	☐
**T2-FLAIR hypointense lesion volume**	☐	☐	☐	☐

4. 3. Should the following **longitudinal** T2-FLAIR **hypointense** lesion measurements be included in the QReport? Please select the purpose(s), **multiple answers per row are possible.** *

Tick all that apply.

	Diagnosis	Monitoring	Prognosis	Not relevant	I don't know
**T2-FLAIR hypointense count (new)**	☐	☐	☐	☐	☐
**T2-FLAIR hypointense volume (change)**	☐	☐	☐	☐	☐

SPINAL CORD LESIONS, OPTIC NERVE LESIONS and PARAMAGNETIC RIM LESION MEASUREMENTS

The following questions are **new questions** about spinal cord lesions and atrophy measurement measurements that should be included in the QReport. Please leave any additional comments about a specific question in the comment box at the end of the section and note the question number.

5. 4. Should the following **cross-sectional focal** lesion measurements in the **spinal cord** be included in the QReport? Please select the purpose(s), **multiple answers per row are possible.** *

Tick all that apply.

	Diagnosis	Prognosis	Not relevant	I don't know
**lesion count**	☐	☐	☐	☐
**Relative lesion count contextualised with MS reference population**	☐	☐	☐	☐

6. 5. Please select which of the following cross-sectional spinal cord **lesion** locations would be of interest for the QReport. **multiple answers are possible.** *

Tick all that apply.

- ☐ Spinal cord level (C2/C3 etc.)
- ☐ Axial description (Lateral/Anterior)
- ☐ None of the above
- ☐ I don't know
- ☐ Other: ____________________

7. 6. Should the following **cross-sectional Gd-enhancing** lesion measurements in the **spinal cord** measurements be included in the QReport? Please select the purpose(s), **multiple answers per row are possible.** *

Tick all that apply.

	Diagnosis	Prognosis	Not relevant	I don't know
**Lesion count**	☐	☐	☐	☐
**Proportion of T2 lesions that show enhancement**	☐	☐	☐	☐

8. 7. Please select which of the following cross-sectional **Gd-enhancing** lesion locations in the **spinal cord** would be of interest for the QReport. **multiple answers are possible.** *

Tick all that apply.

- ☐ Spinal cord level (C2/C3 etc.)
- ☐ Axial description (Lateral/Anterior)
- ☐ None of the above
- ☐ I don't know
- ☐ Other: ____________________

9. 8. Should the following **cross-sectional** and **longitudinal optic-nerve** lesion measurements be included in the QReport? Please select the purpose(s), **multiple answers per row are possible.** *

Tick all that apply.

	Diagnosis	Prognosis	Monitoring	Not relevant	I don't know
**Lesion count**	☐	☐	☐	☐	☐
**Lesion length**	☐	☐	☐	☐	☐
**Lesion laterality**	☐	☐	☐	☐	☐
**Lesion location**	☐	☐	☐	☐	☐
**Lesion count (new)**	☐	☐	☐	☐	☐
**Increase in lesion length**	☐	☐	☐	☐	☐
**New lesion location**	☐	☐	☐	☐	☐

10. 9. Should **optic nerve** be included as an additional/separate lesion location (next to e.g. periventricular/juxta-cortical etc.) in the QReport. **multiple answers are possible.** *

Mark only one oval.

- Strongly Disagree
- Disagree
- Neutral
- Agree
- Strongly Agree
- I don't know
- Other: ____________________

11. 10. Should the following **cross-sectional** and **longitudinal paramagnetic rim lesion (PRL)** measurements be included in the QReport? Please select the purpose(s), **multiple answers per row are possible.** *

Tick all that apply.

	Diagnosis	Prognosis	Monitoring	Not relevant	I don't know	Not applicable, I don't use SWI (or other approriate sequences)
**Lesion count**	☐	☐	☐	☐	☐	☐
**Lesion location**	☐	☐	☐	☐	☐	☐
**New lesion count**	☐	☐	☐	☐	☐	☐
**New lesion location**	☐	☐	☐	☐	☐	☐

12. 11. Should the **central vein sign (CVS)** (present/not present) be included in the QReport. *

Mark only one oval.

- ( ) Strongly disagree
- ( ) Disagree
- ( ) Neutral
- ( ) Agree
- ( ) Strongly agree
- ( ) I don't know
- ( ) Other: ______________________

13. 12. Should the following **longitudinal** **focal** lesion in the **spinal cord** measurements be included in the QReport? Please select the purpose(s), **multiple answers per row are possible.** *

Tick all that apply.

	Diagnosis	Monitoring	Prognosis	Not relevant	I don't know
**Lesion count (new)**	☐	☐	☐	☐	☐
**Relative new lesion count contextualised with MS reference population**	☐	☐	☐	☐	☐

14. 13. Should the following **longitudinal Gd-enhancing** lesion measurements in the **spinal cord** measurements be included in the QReport? Please select the purpose(s), **multiple answers per row are possible.** *

Tick all that apply.

	Diagnosis	Prognosis	Monitoring	Not relevant	I don't know
**Lesion count (new)**	☐	☐	☐	☐	☐
**Proportion of T2 lesions that show enhancement (new)**	☐	☐	☐	☐	☐

15. 14. Which of the following **spinal cord** atrophy measures would you like to see in a **cross-sectional** and **longitudinal** Qreport in a clinical setting? **multiple answers per row are possible** *

Tick all that apply.

	Diagnosis	Prognosis	Monitoring	Not relevant	I don't know
**Mean upper cervical cord area (MUCCA)**	☐	☐	☐	☐	☐
**Cervical cord volume**	☐	☐	☐	☐	☐
**Total cord volume**	☐	☐	☐	☐	☐
**MUCCA change (mm2/year)**	☐	☐	☐	☐	☐
**cervical cord volume change (mm3/year)**	☐	☐	☐	☐	☐
**total cord volume change (mm3/year)**	☐	☐	☐	☐	☐

16. Do you have any remaining comments about the previous questions? *

____

17. 15. Many commercial QReports require both 3D FLAIR and 3D T1-weighted input. Considering the additional scanning time and costs, would you be willing to acquire a 3DT1 alongside the routinely acquired FLAIR? *

Mark only one oval.

- Yes
- No, only 3D FLAIR
- No, only 3D FLAIR and 2D T1
- No, only 2D FLAIR and 2D T1
- No, only 2D FLAIR and 3D T1
- I don't know
- Other: ______________________

18. 16. Should commercial Qreports requiring only 3D-FLAIR-input be developed? *

Mark only one oval.

- Strongly Disagree
- Disagree
- Neutral
- Agree
- Strongly Agree
- Other: ______________________

19. 17. What would you prefer to do when the quality of the segmentation and/or volumetry result is insufficient? *

Tick all that apply.

- [ ] Reject all the measurements
- [ ] Reject specific measurement based on your judgement
- [ ] Manually adjust the segmentation
- [ ] I don't know
- [ ] Only manually adjust the lesion segmentation(s)
- [ ] Other: ______________________

20. 18. Have you already implemented a **commercial** Qreport? If so, please indicate from which company and the usage-frequency. The results will be processed confidentially and both the product name and your name will remain anonymous. *

____

____

____

____

____

21. 19. What have been the bottlenecks in using commercial QReports (e.g. too much time, I don't know how to interpret it)? The results will be processed confidentially and both the product name and your name will remain anonymous. *

____

____

____

____

____

22. Do you have any additional comments/questions about the questionnaire? *

This content is neither created nor endorsed by Google.

Google Forms
